# Recombinant Limb Assay as *in Vivo* Organoid Model

**DOI:** 10.3389/fcell.2022.863140

**Published:** 2022-04-26

**Authors:** Roberto Damián García-García, Estefanía Garay-Pacheco, Jessica Cristina Marín-Llera, Jesús Chimal-Monroy

**Affiliations:** Departamento de Medicina Genómica y Toxicología Ambiental, Instituto de Investigaciones Biomédicas, Universidad Nacional Autónoma de México, Ciudad Universitaria, Ciudad de México, México

**Keywords:** organoid, cell differentiation, patterning, recombinant limbs, limb development, limb organoid

## Abstract

Organ formation initiates once cells become committed to one of the three embryonic germ layers. In the early stages of embryogenesis, different gene transcription networks regulate cell fate after each germ layer is established, thereby directing the formation of complex tissues and functional organs. These events can be modeled *in vitro* by creating organoids from induced pluripotent, embryonic, or adult stem cells to study organ formation. Under these conditions, the induced cells are guided down the developmental pathways as in embryonic development, resulting in an organ of a smaller size that possesses the essential functions of the organ of interest. Although organoids are widely studied, the formation of skeletal elements in an organoid model has not yet been possible. Therefore, we suggest that the formation of skeletal elements using the recombinant limb (RL) assay system can serve as an *in vivo* organoid model. RLs are formed from undissociated or dissociated-reaggregated undifferentiated mesodermal cells introduced into an ectodermal cover obtained from an early limb bud. Next, this filled ectoderm is grafted into the back of a donor chick embryo. Under these conditions, the cells can receive the nascent embryonic signals and develop complex skeletal elements. We propose that the formation of skeletal elements induced through the RL system may occur from stem cells or other types of progenitors, thus enabling the study of morphogenetic properties *in vivo* from these cells for the first time.

## Introduction

During embryonic development, many developmental pathways orchestrate the formation of organs in time and space. In the early stages of embryogenesis, the cell differentiation potential restricts as the pluripotent embryonic cells give rise to three germ layers: ectoderm, mesoderm, and endoderm (Chan et al., 2017; Kumar et al., 2021). Lineage-specific gene regulatory programs within each germ layer activate and coordinate the steps needed for that group of cells to develop into the cell fates required to form tissues and, finally, functional organs. Concomitant with organogenesis is the appearance of stem cells involved in the homeostasis, repair, and regeneration of adult tissues *in vivo* (Slack, 2008). Stem cells are undifferentiated cells with the ability to reproduce themselves (i.e., self-renew to maintain their cell population) and also to give rise to a range of distinct, specialized cells (i.e., differentiate into multiple cell types as needed) (Slack, 2008; Huang et al., 2021; Shu et al., 2021). Stem and progenitor cell research has led to breakthrough developments in regenerative medicine, including stem cell-based therapies for various diseases (e.g., leukemia, aplastic anemia, osteopetrosis) (Río et al., 2018; Almohsen and Al-Mudallal, 2020; Capo et al., 2020). Likewise, this knowledge has created new research fields, such as the generation of organoids. These three-dimensional (3D) structures can be derived from induced pluripotent stem cells (iPSCs), embryonic stem cells (ESCs), or adult stem cells (ASCs). These cells self-organize following the developmental pathways of embryonic development, giving rise to an organ of a smaller size capable of performing its essential functions (Rossi et al., 2018).

Therefore, understanding developmental pathways during embryonic development enables extrapolating those into protocols to induce stem or progenitor cells toward forming an organoid. For many years, the disaggregate-reaggregates of organs or cultures from organ rudiments were used to understand cell differentiation and organogenesis involving soluble factors, cell-cell, and cell-extracellular matrix (ECM) interactions. However, these culture models originated from embryonic or fetal cells from the proper organ in formation or already established (Taketo and Koide, 1981; Escalante-Alcalde and Merchant-Larios, 1992; Saxén and Thesleff, 2007). In recent years, various protocols have been developed to direct stem or progenitor cells to develop into intestine, brain, liver, kidney organoids, among others (Lancaster et al., 2013; McCracken et al., 2014; Dye et al., 2015; Huch et al., 2015; Zhou et al., 2021). However, how to properly create complex structures formed by different cell types from distinct embryonic germ layers (e.g., limbs) has not been determined.

To study cell interactions between mesenchymal cells and the ectodermal cover of developing limb buds, E. Zwilling designed the recombinant limb (RL) assay system (Zwilling, 1964). The RL technique assembles the dissociated-reaggregated or undissociated mesoderm of a limb into an embryonic ectoderm cover and then grafting it into the back of a donor embryo. Notably, the embryonic signals provided by the ectoderm induce gene expression in a spatiotemporal manner driving the 3D organization of a limb-like structure by recapitulating the developmental programs that occur during limb development. Although this model has been mainly used to understand chicken limb development, different approaches have also been reported, including interspecies grafting (Fernandez-Teran et al., 1999), the use of different combinations of mutant and wild-type mesoderm or ectoderm (Kuhlman and Niswander, 1997), or limb mesodermal cells modified by electroporation (Marín-Llera et al., 2021). From these, it is evident that the RL experimental model is adaptable to diverse scenarios. This experimental system has enormous potential to explore the ability of different sources of stem or progenitor cells to generate a limb-like structure.

In this perspective, we discuss the potential of the RL system to generate limbs by recapitulating limb development initiated by embryonic ectodermal signals. We propose that the formation of RLs from iPSCs, ESCs, ASCs, and other progenitor cells can result in a robust *in vivo* organoid model.

## Modeling Organoids to Understand Organ Formation

Organs originate during embryonic development from different tissues to form a specialized unit that performs a particular function (Montell, 2008). An organoid is a small, 3D mass that arises by the self-organization and differentiation of stem or progenitor cells generating the substructures and functions characteristic of the organ of interest. Organoids are generated *in vitro* by inducing the differentiation of stem or progenitor cells as it occurs *in vivo* during embryonic development. Organoid models provide a greater understanding of the cellular and molecular basis of organ development, such as cell differentiation, tissue patterning, developmental timing, regulatory gene expression, and size control.

The potential use of organoid generation lies in exploring tissue repair and disease mechanisms, drug testing, tissue homeostasis, regenerative medicine, and developmental biology at the organ level in a scaled model.

While Hans Clever originally coined the concept of “organoid” (Sato et al., 2009), Yoshiki Sasai and his group were the first to demonstrate that, after the induction of developmental programs, mouse and human ESCs generate 3D complexes composed of organized substructures. They first constructed 3D forebrain models followed by other neural organoids (Eiraku et al., 2008, 2011; Osakada et al., 2009; Kamiya et al., 2011).

Intestinal villi-like structures and crypts were the first organoids generated by inducing individual ASCs (Lgr5+) to organize in 3D suspension, giving rise to distinct cell types such as enterocytes, goblet cells, Paneth cells, and endocrine cells (Sato et al., 2009). Since then, various organoids generated from ASCs have been reported, including the liver and kidney (Lancaster et al., 2013; McCracken et al., 2014; Dye et al., 2015; Huch et al., 2015).

The methodologies used to generate an organoid vary according to which cell is best suited to initiate the process. Sequential induction steps recapitulate development to create an organoid that exhibits the desired and required characteristics. Usually, the generation of an organoid *in vitro* consists of isolating a homogeneous cell population capable of further differentiation (e.g., ASCs, ESCs, iPSCs, other progenitor cells). Next, a matrix rich in proteins, growth factors, and other culture components promotes the proliferation, adherence, and differentiation of cells seeded in a defined substrate (reviewed by Corrò et al., 2020). The most used matrix is Matrigel, a mixture of complex ECM basement membrane components. This gel-like substance is obtained from mouse tumors expressing laminin, nidogen, collagen IV, and heparan sulfate proteoglycans (Kleinman and Martin, 2005). Matrigel components allow embedded cells to execute cellular functions relevant to tissue formation (Rossi et al., 2018). Under these conditions, morphogens and growth factors regulate cells to acquire a proper cellular fate, guiding them to self-organize. During organoid formation, cells need determined physiological conditions to commit to specific-tissue cell types and develop into a 3D organized structure according to specific developmental programs (Ehrmann and Robert, 1956; Murry and Keller, 2008; Sasai, 2013). Other forms of generating organ constructs have been elaborated using bioengineering, organs-on-a-chip, and *in situ* approaches, including gene editing, interspecies chimeras, and cellular reprogramming (reviewed in Xia and Izpisua Belmonte, 2019).

Although organoids are an invaluable strategy for modeling early tissue organization characteristics *in vivo*, they have some limitations. Organoids do not fully mimic the physiological organ as they lack some tissue components, vascularization, and immune cells, limiting organ maturation and resulting in incomplete function (de Souza, 2018). Organoids are built lacking vascularization, an essential feature of all tissues to supply nutrients and allow for adequate perfusion. It may result in atypical physiology of the organoid compared to the organ to be modeled. Advances in different strategies to vascularize organoids are reviewed by Strobel et al., 2022. Furthermore, using different sources to establish organoid cultures, the heterogeneity of progenitors and differentiated cells may affect organoid formation that not necessarily corresponds to the *in vivo* counterparts.

The *in vivo* environment is complex, with multiple cell-cell and cell-matrix interactions giving rise to diverse signaling networks that dynamically change according to organ homeostasis. It is expected that attempting to model this complexity *in vitro* will be challenging.

## Advances in Limb Organoid Generation

One challenge in generating an organoid is the complexity of the organ of interest. The more complex the organ, the more difficult is the generation of the organoid. Organoid formation involves driving cell populations to spatially organize into a functional structure through exposure to morphogens and specific differentiation signals. Thus, a challenge for the generation of a limb organoid is to mimic tissue organization and function. Limb formation is a powerful model for investigating cell differentiation during development because it involves the establishment of a 3D pattern that directs the morphogenesis, shaping, and positioning of each tissue (McQueen and Towers, 2020; Royle et al., 2021). Limb buds emerge at specific positions along the flank of the embryo (Hamburger and Hamilton, 1992; Feneck and Logan, 2020). The limb bud is formed by undifferentiated mesenchymal cells derived from the lateral plate mesoderm (LPM) with an ectodermal epithelium covering these mesenchymal cells. Tissues differentiate into the limb bud in response to signals from different signaling centers that control the proximodistal [(PD), shoulder to fingers], anteroposterior [(AP), thumb to finger], and dorsoventral [(DV), from the back of the hand to palm] axes. The apical ectodermal ridge (AER) is the thickened epithelium located at the most distal limb ectoderm and controls the PD axis. Cells from the AER express *Fgf8*, which along with *Wnt3a*, maintains the mesodermal cells underneath the AER in an undifferentiated, proliferative state (Ohuchi et al., 1997; ten Berge et al., 2008). As the limb bud grows, the undifferentiated cells underneath the AER begin to differentiate toward the chondrogenic and tenogenic lineages when they stop receiving signals from the AER (Dollé et al., 1989; Roselló-Díez et al., 2014; Marín-Llera et al., 2019; Marin-Llera et al., 2021). The zone of polarizing activity (ZPA) controls limb bud AP polarity and is located at the posterior margin of the limb bud. When the ZPA is grafted to the anterior zone of a limb bud, it induces mirror-image digit duplications. This signaling center is characterized by *Sonic hedgehog* (*Shh*) expression (Riddle et al., 1993; Fujii et al., 2021; Gamart et al., 2021). Finally, the ventral and dorsal limb bud ectoderms specify DV polarity. *Engrailed 1 (En-1)*, a transcription factor that specifies the ventral ectoderm, and *Wnt7a,* which specifies the dorsal ectoderm by inducing *Lmx1* gene activation, give rise to the DV phenotype of a limb (Loomis et al., 1996; Soshnikova et al., 2003; Haro et al., 2014). These three signaling centers are essential for patterning limbs, committing mesodermal cells to different lineages, and coordinating them within the limb to give rise to the appendicular skeletal system.

One methodology for evaluating the differentiation of limb bud progenitor cells is high-density primary limb mesenchymal culture, also known as a micromass (MM) culture (Ahrens et al., 1977). In MM cultures, the limb mesenchymal cells recapitulate the developmental process observed in limb development to give rise to cartilage cells during the formation of skeletal elements. Limb bud mesenchymal cells condense and aggregate to form 3D cartilage nodules. In long-term MM cultures, the cartilage nodules provide matrix calcification accompanied by increased alkaline phosphatase activity (Arzate et al., 1996; Mello and Tuan, 1999). Although MM cultures help study the basic mechanisms underlying the differentiation of limb bud cells and their regulation, this method has a limited ability to recapitulate the assembly of progenitors into organized tissues that span the entire limb.

Another model used to understand limb formation is the *ex vivo* limb bud culture system (Fell and Robison, 1929). At a particular stage in limb bud development, the bud contains all the elements required to develop autonomously. In this system, the embryonic limb is sectioned and placed in culture media to continue forming skeletal structures with the proper 3D organization of a mature limb, preserving all the cell-cell and cell-ECM interactions that control cell differentiation and morphogenesis (Hall, 1981; Smith et al., 2013). This methodology facilitates the study of the molecular mechanisms regulating chondrocyte differentiation (Schnabel et al., 2006; Lorda-Diez et al., 2010), toxicology testing, and aberrant embryonic limb development (Yan and Hales, 2019, 2021). Although *ex vivo* limb bud cultures provide a tool to understand skeletal and limb development, the initial commitment processes required to enter particular differentiation states remain hard to study. On the other hand, mesenchymal stromal cells (MSCs), a multipotent population obtained from various fetal and adult tissues, differentiate into osteogenic, chondrogenic, and tenogenic lineages mainly for regenerative medicine applications (Toh et al., 2017; Xia et al., 2018; Jiang et al., 2020). MSCs’ potential to differentiate into limb lineages has been investigated mainly using 2D cultures systems.

Obtaining limb-bud-like mesenchymal (LBM) cells from iPSCs or ESCs to create limb organoids is essential to recapitulate early commitment events during embryonic development. Cell differentiation of stem or progenitor cells into LBM cells can be driven by specification signals from the middle primitive streak (midPS) and the LPM. Providing induced LBM cells with the inductive signals necessary to trigger step-by-step their commitment and differentiation into a specific cell lineage ought to result in the formation of a limb organoid. One approach to inducing an LPM state relies on the transient transfection of miRNAs. In mouse ESCs, transient co-transfection of mmu-miR-126a-3p, mmu-miR-335-5p, and mmu-miR-672-5p promotes differentiation toward LPM lineages, thereby increasing the number of LPM-like cells (Tuysuz et al., 2021). Furthermore, miR-199a-3p, miR-214-3p, and miR-483-3p are enriched in mesodermal cells differentiated from human ESCs. However, their roles in specifying the mesoderm into different tissue subtypes have not yet been fully characterized (Ishikawa et al., 2017). Loh et al. (2016) established a developmental roadmap to direct the commitment of cell lineages to particular fates. The reprogramming of human adult somatic cells into human-induced pluripotent stem cells (hiPSCs) by promoting the expression of four transcription factors, including Oct4/Sox2/c-Myc/KLF4 or Oct4/Sox2/NANOG/LIN28, has also been reported (Takahashi and Yamanaka, 2006; Takahashi et al., 2007; Yu et al., 2007). Loh et al. (2016) demonstrated how to generate limb precursor cells after treating stem cells with factors that progressively regulate the formation of mesodermal lineages. They treated hiPSCs with activin, BMP4, CHIR99021, and FGF2 to induce a mid-primitive streak-like (midPS-like) state (Loh KM., 2016). Then, the administration of an ALK5 inhibitor, BMP4, and a Wnt antagonist directs the midPS-like cells down an LPM-like path (Loh et al., 2016). Thereafter, progenitor cells can differentiate into specific cell lineages. Differentiating hiPSCs into LBM cells requires a different combination of chemicals that modulate WNT, BMP, TGF-β, and hedgehog (HH) signaling added to the cells in the appropriate times resulting in PRRX1^+^ (cell-specific marker) LBM cells (Yamada et al., 2021). In addition to the Yamaka factors, another study used *Prdm16, Zbtb16, and Lin28*, generally expressed in the embryonic limb bud, to reprogram mouse non-limb fibroblast into LBM progenitors (Atsuta et al., 2021). In other experimental approaches, hiPSCs seeded into high-density MM cultures treated for 21 days with BMP-2 recapitulate the osteochondrogenic transcriptional network and differentiate to the chondrogenic lineage, including articular cartilage, transient cartilage, and fibrocartilage (Guzzo et al., 2013).

One of the most recent attempts to create a limb organoid was reported by Mori et al. (2019). A polarized limb-like structure was generated from aggregates of mouse embryonic stem cells (mESCs) cultured in a scaffold of Matrigel and treated with BMP4 and retinoic acid. While the expression of *Tbx4, Tbx5, Hand2, Irx3, Meis1, and Meis2,* genes involved with the induction, organization, and establishment of limb buds were found, these 3D cultures failed to generate a structure similar to the AER and, therefore, neither recapitulated the differentiation process nor the morphogenetic patterning observed during limb development.

These approaches highlight the importance of understanding the embryonic signals involved in limb development to guide the differentiation of undifferentiated cells into limb-like structures, demonstrating that the sequential activation of developmental programs is necessary for inducing differentiation into specific lineages. Undoubtedly, these works have increased our knowledge of limb organogenesis by generating limb organoids. However, these protocols still fail to recapitulate the morphogenetic processes required to form complex skeletal structures.

## Recombinant Limb Assay as a Tool for Limb-Organoid Generation

Edgar Zwilling (Zwilling E., 1964) developed the recombinant limb (RL) assay system to understand the interactions between limb bud ectoderm and mesenchymal cells. This technique, usually used in avian species, consists of assembling whole or dissociated-reaggregated mesodermal cells into an ectodermal cover obtained from an early limb bud to then graft into the dorsal part of a donor chick embryo (for a detailed protocol, see Marín-Llera et al., 2022, Ros et al., 2000). Patterning signals from the embryonic ectoderm induce cell differentiation of mesodermal cells in a spatial-temporal manner to form a limb-like structure following the developmental programs as occurs during limb development. This phenomenon proves that mesodermal limb cells lose their positional identity within the limb bud once they are dissociated. However, they remain competent to ectodermal signals in the RL system, re-specifying its positional values, differentiating, and generating recognizable limb structures (Zwilling E., 1964). Morphogenetic processes of the RL are enhanced by adding an intact ZPA mesoderm at one of the ectodermal edges (MacCabe et al., 1973; Crosby and Fallon, 1975; Frederick and Fallon, 1982). In this way, the RL system provides the spatial-temporal signals mimicking the embryonic limb bud allowing mesodermal cells to differentiate and pattern. RL system provides the PD signals (*Fgf8 and Fgf4*) and DV signals (*En-1* and *Wnt7a*) of the ectoderm, promoting *Fgf10*, *Lmx-1,* and *Shh* expression in mesodermal cells (Kuhlman & Niswander, 1997; Elisa Piedra et al., 2000). The expression of patterning genes promotes positional information within RL mesodermal cells such as *Msx1, Msx2, Hoxd11, Hoxd12, and Hoxd13* (Ros et al., 1994; Cooper et al., 2011; Rosello-Diez et al., 2011). Twenty-4 hours after grafting RL, cells start the differentiation programs by committing to chondrogenic lineages by expressing *Sox9*, and muscle progenitors migrate inward RL from the somites expressing *MyoD* and *Pax3* (Cooper et al., 2011). Interestingly, in the RL assay, the patterning of these progenitors resembles normal development when grafted in the somite area with high levels of retinoic acid (Fernandez-Teran et al., 1999; Cooper et al., 2011). Thus, the RL system recapitulates the differentiation, morphogenesis, and patterning programs observed in normal limb development.

One of the advantages of the RL system is the variety of combinations between its elements. It is well known that the molecular signals among vertebrate limb development are conserved throughout tetrapod evolution. Research groups have adapted Zwilling’s technique to prove that cells from different species, including the turtle (Fallon & Simandl, 1984) and mouse (Kuhlman & Niswander, 1997), can interpret the signals emitted from an ectodermal jacket that is not their own. Moreover, mesenchymal cells from the anterior or posterior limbs can be placed inside the anterior or posterior ectoderm (Crosby and Fallon, 1975; Frederick and Fallon, 1982). Some points to consider about this technique are the graft efficiency that may vary between mesodermal sources and the high number of chicken embryos needed to generate the RL (mesoderm donors, ectoderm donors, and host embryos). Also, fine manipulations are needed in each step to guarantee the technique’s success. Furthermore, understanding the formation of tendons and vasculature in RLs is not yet fully elucidated.

Based on these characteristics the RL model is a well-suited to evaluate the biology of stem and progenitor cells isolated directly from an organism (e.g., LBM cells). The RL system’s versatility permits the creation of multiple combinations of cells from different sources, developmental stages, or positions along the limb, whole (undissociated), or reaggregated cells, even until modified LBM cells overexpress distinct molecules, as shown by Marín-Llera et al., 2021.

**FIGURE 1:**
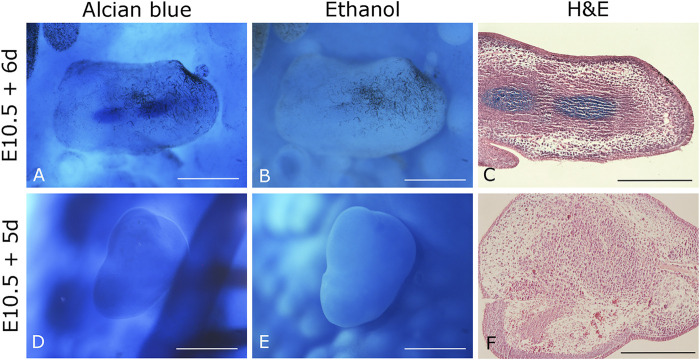
Chimeric mouse-chicken recombinant limbs. **(A–C)** Six-day recombinant limbs. Limb bud mesodermal cells from 10.5 *dpc* mouse embryos were assembled in chicken ectoderms. **(D–F)** Five-day recombinant limbs. Limb bud mesodermal cells from 10.5 *dpc* mouse embryos were ensembled in chicken ectoderms. Chicken ectoderms and host embryos were obtained from the 22 HH stage. Data represent two independent experiments. Scale bar 100 µm.

**FIGURE 2:**
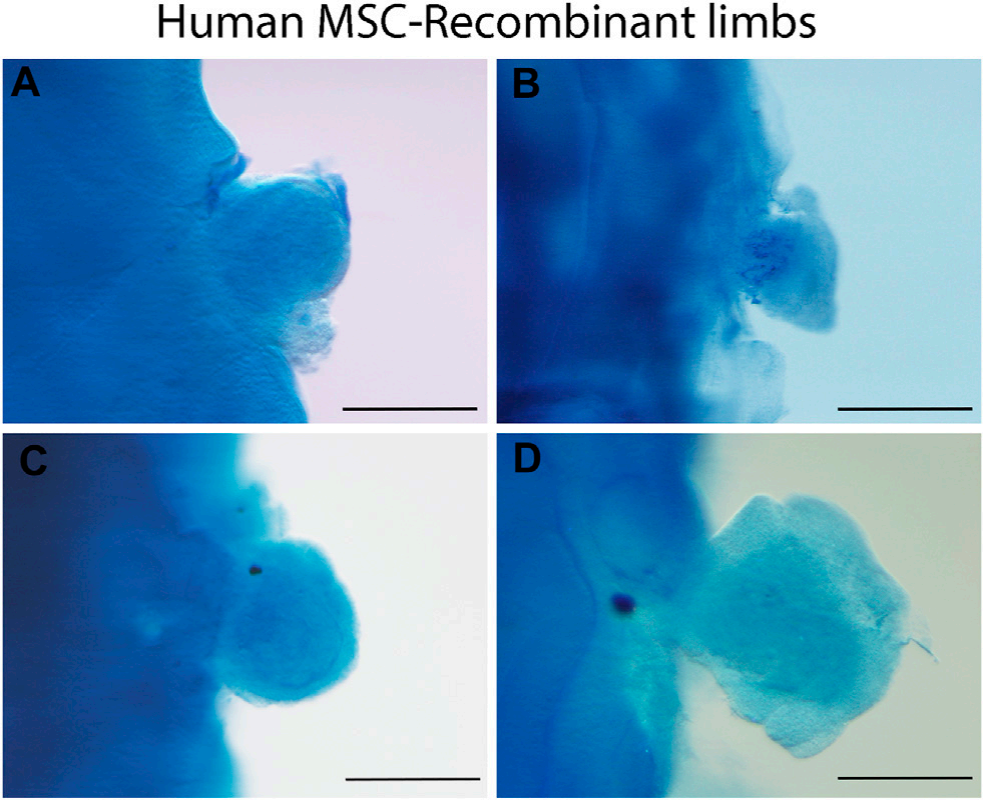
Human MSC-derived RL from different sources. Alcian blue staining of 24 h recombinant limbs performed with human MSCs assembled in chicken ectoderms. MSCs were obtained from **(A)** bone marrow, **(B)** Wharton’s jelly, **(C)** umbilical cord blood, and **(D)** placenta. Chicken ectoderms and host embryos were obtained from the 22 HH stage. Scale bar 100 µm.

Data from our laboratory has demonstrated that mouse limb mesodermal cells are competent to receive chicken ectodermal signals and form skeletal elements after 6 days. Skeletal elements are organized similarly as it occurs during limb development (Figures 1A–C). On the other hand, in the 5-day mouse-chicken RL, mouse mesodermal cells are condensed in the center, and no skeletal elements are observed (Figures 1D–F). This suggests that the specification of mouse mesodermal cells in this system is delayed relative to the chicken-chicken RL. The successful generation of a chimeric RL using mouse mesoderm demonstrates the possibility of combining cells from different mammalian species, thereby creating opportunities to study morphogenesis, patterning cell-cell interactions, cell migration, and cell differentiation at the cellular and molecular levels on these cells. Notably, the source of the mesodermal component of the RL is not limited to limb cell sources. Our unpublished work demonstrated that RL could be obtained from previously expanded cells with mesodermal differentiation capacity as adult MSCs. MSCs from bone marrow (BM), Wharton’s jelly (WJ), umbilical cord blood (UCB), and placenta (P) placed under the embryonic ectodermal signals successfully formed RL after 24 h after grafting (Figure 2). Interestingly, MSCs are committed and spatially organized differently under these conditions depending on their origin (data not shown). These data suggest that it might be possible to generate RL from other ASCs and PSCs or stem or progenitor cells (e.g., ESCs, iPSCs) induced through *in vitro* differentiation protocols. The RL system permits us to evaluate cells’ behavior in response to embryonic patterning signals and, even more relevant, to study their capacity to interpret morphogenetic signals that could lead them to form the pattern of limb bud skeletal elements *in vivo*.

The RL system exposes cells simultaneously to signaling centers, thereby physiologically mimicking the limb microenvironment and allowing undifferentiated cells to develop into distinct limb cell types simultaneously by synthesizing both the ECM and tissue-specific proteins. In addition to individual cell differentiation, groups of cells organize into a complex structure with a 3D pattern. These are essential characteristics required for a system to be considered an organoid. In this sense, the RL assay represents a powerful *in vivo* system for generating limb organoids.

## Conclusion

Cell differentiation and morphogenesis are fine-tuned processes that lead to the formation of specialized cell types, organized tissues, and functioning organs directing cell fate from embryonic development to an independent living organism. Understanding the early cell differentiation steps is essential to model these complex processes in an experimental setting. The RL system is a powerful experimental model for studying patterning, morphogenesis, cell-cell interactions, cell migration, commitment, and cell differentiation at the cellular and molecular levels. Currently, the use of the RL model is restricted to limb developmental biology. However, this infrequently used technique represents an *in vivo* organoid system. It conserves the expression of the ectodermal limb bud signaling centers that mediate the morphogenesis and commitment of undifferentiated cells along distinct developmental paths. This model also faithfully maintains the appropriate gene expression patterns with spatial-temporal accuracy. The RL model establishes the proper 3D polarization of limb-like structures and recapitulates the differentiation programs observed during development, resulting in correctly positioned skeletal elements. Furthermore, we consider that the RL assay system permits countless applications across numerous biological questions without being restricted to limb developmental biology and the use of LBM cells.

## Data Availability

The original contributions presented in the study are included in the article/Supplementary Material, further inquiries can be directed to the corresponding authors.
